# Prenatal diisononyl phthalate exposure induces the development of pulmonary dysplasia in offspring

**DOI:** 10.1038/s41598-026-50939-8

**Published:** 2026-05-03

**Authors:** Dan Li, Chengyu Zhao, Fu Xu, Yao Yuan, Sihan Zhang, Yuanyuan Tian, Meiqiong Wu

**Affiliations:** 1 Shanxi College of Technology School of Materials Engineering, Shuozhou, 036000 Shanxi China; 2 Shanxi Medical University School of Public Health, Taiyuan, 030001 Shanxi People’s Republic of China; 3 Shanxi Provincial Cancer Hospital, Taiyuan, 030006 Shanxi China; 4 Grand Hospital of Shuozhou, Shuozhou, 036000 Shanxi China; 5 Changzhi Hospital of Traditional Chinese Medicine, Changzhi, 046000 Shanxi China

**Keywords:** Prenatal DINP exposure, Pulmonary fibrosis, Hypoalveolarization, IL-17A, Developmental biology, Diseases, Genetics, Medical research

## Abstract

As a widely used plasticizer, diisononyl phthalate (DINP) has been considered an emerging environmental pollutant. Epidemiological evidence shows that prenatal DINP exposure is linked to an increased risk of lung diseases in offspring and adults. However, little is known about their relationship at the molecular level. We exposed pregnant mice to DINP (100 mg/kg/day) by intragastric administration throughout gestation. On postnatal days (PNDs) 21 and 42, offspring were sacrificed and the lungs were collected. We found that prenatal DINP exposure significantly caused hypoalveolarization in male offspring on PND 21, which was reversed during postnatal development. Moreover, prenatal DINP exposure significantly caused pulmonary fibrosis in male offspring on PND 42. However, neither of these changes was observed in female offspring. KEGG enrichment showed that differentially expressed genes (DEGs) in the lungs of male offspring were closely associated with the calcium signaling and IL-17 signaling pathways. Importantly, decreased *Htr4* expression might contribute to hypoalveolarization, and the elevated *Il-17a* expression might be responsible for prenatal DINP exposure-induced pulmonary fibrosis in male offspring. Our results demonstrated that prenatal DINP exposure had a persistent impact on lung development in male offspring, including early-stage impaired alveolarization and later-stage fibrosis.

**Supplementary Information:**

The online version contains supplementary material available at 10.1038/s41598-026-50939-8.

## Introduction

Bronchopulmonary dysplasia (BPD) is a chronic lung disease of very premature infants characterized by lung inflammation and dysregulation of alveolar and vascular development and is associated with long-term respiratory sequelae^1^. Since lung development begins in utero, studies have shown that prenatal exposure to environmental pollutants may affect early lung growth and development, leading to abnormal lung structure and function in later life^2^. Moreover, some health outcomes, such as BPD, that are caused by exposure to environmental pollutants during fetal lung development are not found in adults following the same exposure^3^. It’s also reported that prenatal exposure to environmental pollutants increases the susceptibility of adult rat offspring to pulmonary fibrosis^4^. These studies suggest that lung diseases in adults may originate in utero.

Phthalates have been used to increase the plasticity of rigid plastics (such as polyvinyl chloride) for more than 50 years, with annual production now exceeding 3 million metric tons, and the impact of phthalate exposure on human health has become an issue of concern^5^. Prenatal exposure to phthalates affects lung development and causes wheezing and asthma in childhood in a sex-dependent manner^2^. Previously, due to the belief that diisononyl phthalate (DINP) has relatively low toxicity, it has been considered an environmentally friendly plasticizer^6,7^. However, recent Swedish Environmental Longitudinal, Mother and child, Asthma and allergy (SELMA) study show that DINP can cross the placenta and interfere with many biological pathways during fetal development^8,9^. Maternal exposure to phthalates in early pregnancy may induce early childhood wheezing, especially in children who do not have asthma^8^. Animal studies have shown that maternal intake of DINP can aggravate ovalbumin-induced airway inflammation in young mice^10^. These studies indicate that prenatal DINP exposure can affect lung development in early life, leading to increased susceptibility of offspring to respiratory diseases in adulthood.

However, little is known about the effects of prenatal DINP exposure on lung development in early life. We hypothesized that prenatal DINP exposure impairs postnatal lung alveolarization and induces perivascular fibrotic remodeling in offspring through sex-dependent alterations in calcium signaling and IL-17–mediated inflammatory pathways.

## Materials and methods

### Animals and prenatal DINP exposure

Six-week-old ICR mice (30 females and 15 males) were purchased from Beijing Vital River Laboratory Animal Technology (Beijing, China). The production license number was: SCXK (Jin) 2019-0004, and the usage license number was: SYXK (Jin) 2019-0007, with all animal protocols approved by the Institutional Animal Care and Use Committee of Shanxi Medical University. When the animal was euthanized, CO_2_ (10%−20%) was slowly introduced to cause the animal to lose consciousness due to oxygen deprivation. After the animal lost consciousness, the CO_2_ concentration was raised to the lethal level (70%), resulting in respiratory arrest and cardiac arrest. The entire process was carried out by trained personnel with extensive research experience. All procedures strictly adhered to relevant Chinese national regulations on laboratory animal welfare and ethics, and complied with the ARRIVE guidelines. Every effort was undertaken to minimize animal suffering and reduce the number of animals utilized.

Mice were maintained under standard conditions as described previously^11^. One male mouse and two female mice per cage were mated. Female mice with a positive vaginal plug were considered pregnant, and the day on which the positive plug was detected was regarded as GD0.5 of gestation. Pregnant mice were randomly divided into the control (*n* = 7) and DINP-exposed groups (*n* = 6), and they were exposed to DINP (100 mg/kg bw, dissolved in corn oil) or corn oil by intragastric administration once every day, starting at GD0.5 and continuing throughout gestation. The detailed selection of the dosage is provided in Text S1 in the Supplementary Material (SM). Offspring were sacrificed to collect the lungs on postnatal days (PNDs) 21 and 42.

### Hematoxylin–eosin (H&E) staining

After dissection, fresh lungs were fixed with 4% paraformaldehyde for 24 h, gradiently dehydrated with ethanol, embedded in paraffin, and sectioned. The slices were dried in an oven at 60℃, deparaffinized in gradients of xylene and absolute ethanol, and stained with H&E (Wuhan Servicebio Technology, Wuhan, China) following the manufacturer’s protocol. Finally, the slices were sealed with neutral resin (Sinopharm Chemical Reagent, Shanghai, China). Mean linear intercept (MLI) was determined on H&E-stained lung sections. Briefly, 10 non-overlapping photos of lung tissues were captured with an Olympus CX43 microscope (Tokyo, Japan) at 200× magnification. The structures of both big vessels and airways were excluded. The photos were analyzed using a coherent system of 21 lines and 42 points embedded in the CellSens software (Olympus, Tokyo, Japan). The area of alveoli and the thickness of alveolar walls were analyzed using Image J (Fiji).

### Immunohistochemistry (IHC) staining

IHC staining was conducted as described previously^11^. The sections were deparaffinized, rehydrated, and processed for antigen retrieval. Then, IHC staining was conducted, and the sections were observed under a light microscope. Detailed information is provided in Text S2 in Supplementary Material (SM).

### Masson’s trichrome staining

Lung tissues were deparaffinized in gradients of xylene and ethanol, stained with hematoxylin (Jiangsu KeyGEN BioTECH, Nanjing, Jiangsu, China) for 5 min, differentiated with 0.1%HCl, and rinsed with tap water until blue. Then, the sections were stained with Masson’s trichrome (Guangzhou Yike Biotechnology, Guangzhou, China) following the manufacturer’s protocol. After staining, the sections were sealed with neutral resin (Sinopharm Chemical Reagent, Shanghai, China) and observed using an Olympus BX63 fluorescence microscope (Olympus, Japan). The quantification of the Marson staining results was performed using ImageJ (Fiji).

### Real-time quantitative reverse transcription PCR (RT-qPCR)

Total RNA was extracted from lung tissues as described previously^12^. The genes of interest included elastin *(Eln)*, lysyl oxidase-like 1*(Loxl1)*, platelet-derived growth factor receptor alpha (*Pdgfr-α*), leucine-rich repeat kinase 2 (*Lrrk2*), *Acta2*, tissue inhibitor of metalloproteinases-1 (*Timp1*), interleukin-6 (*IL-6*), *IL-17a*, *IL-17ra*, connective tissue growth factor (*Ctgf*), transforming growth factor beta *(Tgf-β)*, and 5-Hydroxytryptamine Receptor 4 (*Htr4*). Primer sequences used for RT-qPCR are listed in Table 1. The housekeeping gene *Gapdh* was used as an internal control.

**Table 1:** Primer sequences for RT-qPCR.

Genes	Annealing temperature/℃	Sequence (5ʹ-3ʹ)
Il-17a	48.7	Forward: CCAGGGAGAGCTTCATCTGT
		Reverse: AGGAAGTCCTTGGCCTCAGT
Il-17ra	48.7	Forward: AGATGCCAGCATCCTGTACC
		Reverse: CACAGTCACAGCGTGTCTCA
Pdgfr-α	41.6	Forward: TTCAAGACCAGCGAGTT
		Reverse: GGGCAGCACATTCATAC
Loxl1	51.7	Forward: CCGACCCAACCAGAATG
		Reverse: GTCCTCCAGGCAGAAGC
Eln	43.8	Forward: AAAGCAGGTTTGGGTC
		Reverse: TAGCAGCAGATTTAGCG
Lrrk2	51.2	Forward: GCTATCTTGCATTTCGTTGTGC
		Reverse: CCCAGGATTCCCAATGAACC
Acta2	57.0	Forward: GACGCTGAAGTATCCGATAGAACACG
		Reverse: CACCATCTCCAGAGTCCAGCACAAT
Il-6	57.5	Forward: CAACGATGATGCACTTGCAGA
		Reverse: GTGACTCCAGCTTATCTCTTGGT
Timp1	51.2	Forward: GCAAAGAGCTTTCTCAAAGACC
		Reverse: AGGGATAGATAAACAGGGAAACACT
Ctgf	51.2	Forward: GGGCCTCTTCTGCGATTTC
		Reverse: ATCCAGGCAAGTGCATTGGTA
Tgf-β	55.0	Forward: CGTCAGACATTCGGGAAGCA
		Reverse: TGCCGTACAACTCCAGTGAC
Htr4	54.5	Forward: CGTGGTGTGTCTTCATGGTC
		Reverse: GATTCGGTAATAGGCCAGCA
Gapdh	51.0	Forward: TGGTCCAGGGTTTCTTACTC
		Reverse: GTTGTCTCCTGCGACTTCA

### Transcriptome sequencing analyses

Lung issues were collected from male offspring for mRNA sequencing. RNA extraction, library construction, sequencing, and raw data processing were carried out at Shanghai Biotechnology Company (Shanghai, China) using the protocols described previously^13^. Briefly, total RNA was extracted using TransZol Up Plus RNA Kit (Cat#ER501-01, Trans) according to the manufacturer’s instruction, and RNA integrity was checked using an Agilent Bioanalyzer 2100 (Agilent Technologies, Santa Clara, CA, USA). Total RNA was qualified and purified using RNA Clean XP Kit (Cat#A63987, Beckman Coulter, CA, USA) and RNase-Free DNase Set (Cat#79254, QIAGEN, GmBH, Germany). The library was constructed from the purified total RNA through a series of steps, including mRNA separation, fragmentation, first-strand cDNA synthesis, second-strand cDNA synthesis, end repair, 3’end addition, ligation, and enrichment. The concentration and size of the library were determined using Qubit^®^ 2.0 Fluorometer and Agilent 2100, respectively. The sequencing process was run and monitored using an Illumina HiSeq 2500 System, and real-time data analysis was performed using HiSep Control software. To analyze the differential expression of mRNAs, raw reads were normalized to Fragments per Kilobase of exon per Million mapped reads (FPKM). A p-value of less than 0.05 was used as a screening criterion for identifying differentially expressed genes (DEGs), and the biological pathways of DEGs were analyzed using the Kyoto Encyclopedia of Genes and Genomes (KEGG) database.

### Enzyme-linked immunosorbent assay (ELISA)

Lung tissues were homogenized in ice-cold PBS. The homogenates were centrifuged at 3000 r for 20 min at 4 °C, and the supernatants were collected. The total protein concentration of each supernatant was determined using BSA (Solarbio, Beijing, China). The concentrations of various target proteins, including, 5-Hydroxytryptamine Receptor4 (HTR4) endothelin-1 (ET-1), vascular endothelial growth factor (VEGF), and androgen receptor (AR), were quantified using ELISA kits (Animalunion Biotechnology, Shanghai, China) according to the manufacturers’ protocols.

### Statistical analysis

All data are presented as means ± standard error (SE) and analyzed using GraphPad Prism 10 (GraphPad Software, USA). For data obtained at each time point (PND 21 and 42), statistical differences among different groups were analyzed using one-way analysis of variance (ANOVA), followed by Tukey’s Honestly Significant Difference (HSD) post hoc test for multiple comparisons. *P* < 0.05 was considered statistically significant.

## Results

### Prenatal DINP exposure impairs alveolarization in offspring

**Fig. 1:**
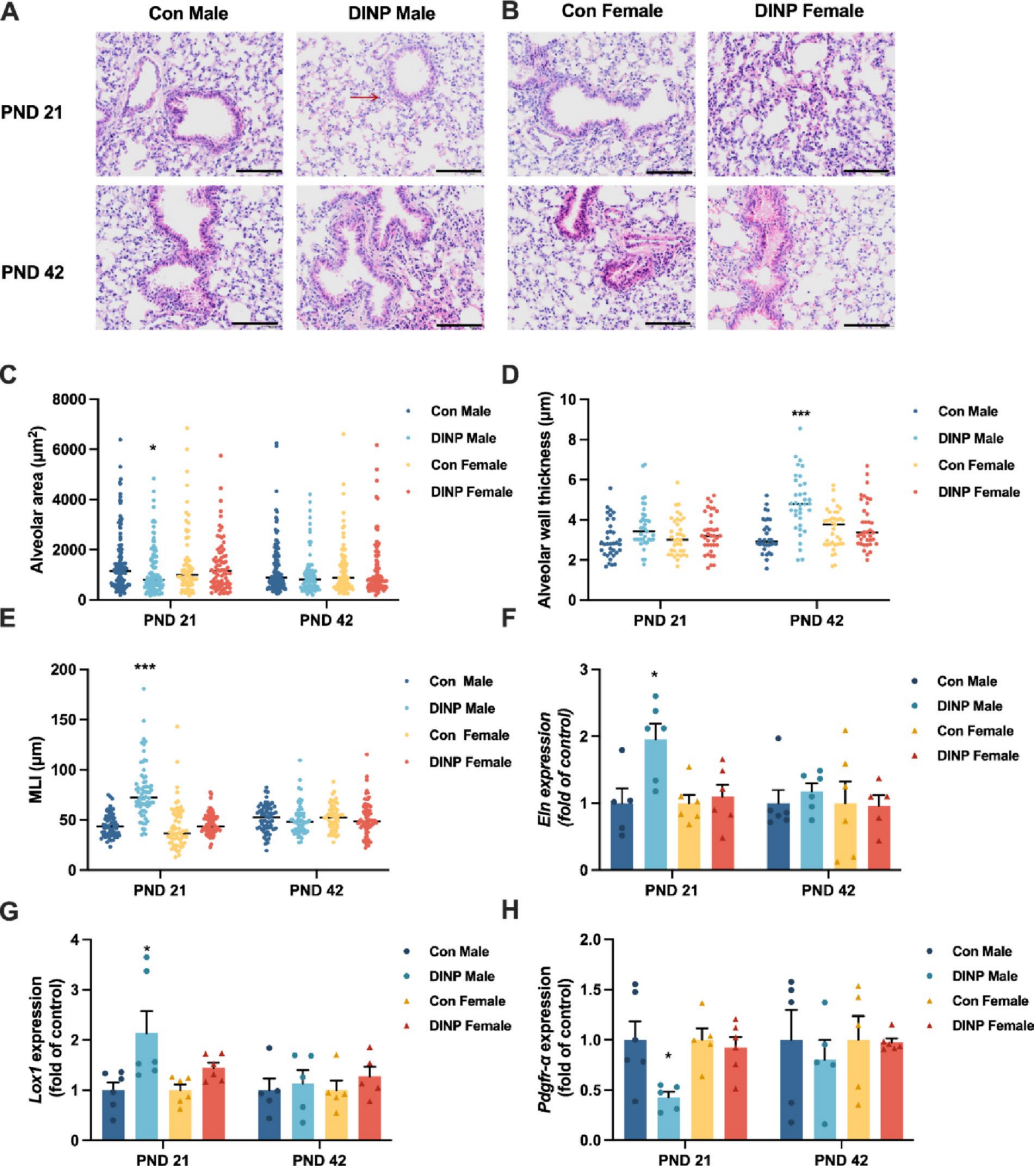
Effects of prenatal DINP exposure on lung alveolar development in offspring. (**A**) Representative images (200×) of H&E-stained lung sections of male offspring on PNDs 21 and 42. Bar = 100 μm (**B**) Representative images (200×) of H&E-stained lung sections of female offspring on PND 21 and 42. Bar = 100 μm (**C**) Alveolar area (*n* = 115 to 120 alveoli for each group, from two offspring of different dams). (**D**) Alveolar wall thickness (*n* = 32 to 37 alveoli for each group, from two offspring of different dams). (**E**) Mean linear intercept (MLI) of alveoli (*n* = 60 to 70 alveoli for each group, from two offspring of different dams). (**F**) mRNA expression of *Eln* (*n* = 5 to 6 for each group, from different dams). (**G**) mRNA expression of *Lox1* (*n* = 5 to 6 for each group, from different dams). (**H**) mRNA expression of *Pdgfr-α* (*n* = 5 to 6 for each group, from different dams). Data are expressed as mean ± SE. *P < 0.05, **P < 0.01, ***P < 0.001, indicates significance between different groups. *Con* control; *DINP* prenatal DINP-exposed offspring.

To determine whether prenatal DINP exposure caused abnormal lung development in offspring, the changes in postnatal lung alveolarization were observed using H&E staining. Compared with the control group, DINP caused hypoalveolariation in male offspring on PND 21 (Fig. 1A). In contrast, hypoalveolarization (impaired alveolar septation) was not as significant in the female offspring as it was in male offspring (Fig. 1B). Quantitative analysis of the alveolar area and the thickness of alveolar walls was conducted. The results showed that the mean alveolar area was significantly decreased in male offspring on PND 21, while the thickness of alveolar walls increased on PND 42 in male offspring after prenatal DINP exposure (Fig. 1C and D). To quantitatively confirm impaired alveolarization, we performed morphometric analyses. The MLI significantly increased in male offspring on PND 21 (Fig. 1E). An increased MLI is a well-established morphometric index of alveolar enlargement and simplification, indicative of impaired alveolarization—a hallmark of disrupted lung development. The combination of increased MLI and decreased alveolar areas is a hallmark of hypoalveolarization rather than destructive emphysema. These quantitative data demonstrate that prenatal DINP exposure causes a persistent deficit in alveolar number in males.

To confirm the changes in the alveoli of male offspring, we profiled several genes (*Pdgfr-*α, *Eln*, and *Loxl1*) encoding alveolar development. On PND 21 following prenatal DINP exposure, the mRNA expression of *Eln* and *Loxl1* in the lungs of male offspring increased significantly (Fig. 1F and G) compared to control male offspring, while the mRNA expression of *Pdgfr-α* decreased significantly (Fig. 1H). The results suggest that prenatal DINP exposure causes a persistent structural deficit in alveolarization in male offspring, along with transient dysregulation of developmental genes.

### Prenatal DINP exposure induces perivascular fibrosis and pulmonary vascular dysfunction in offspring

**Fig. 2:**
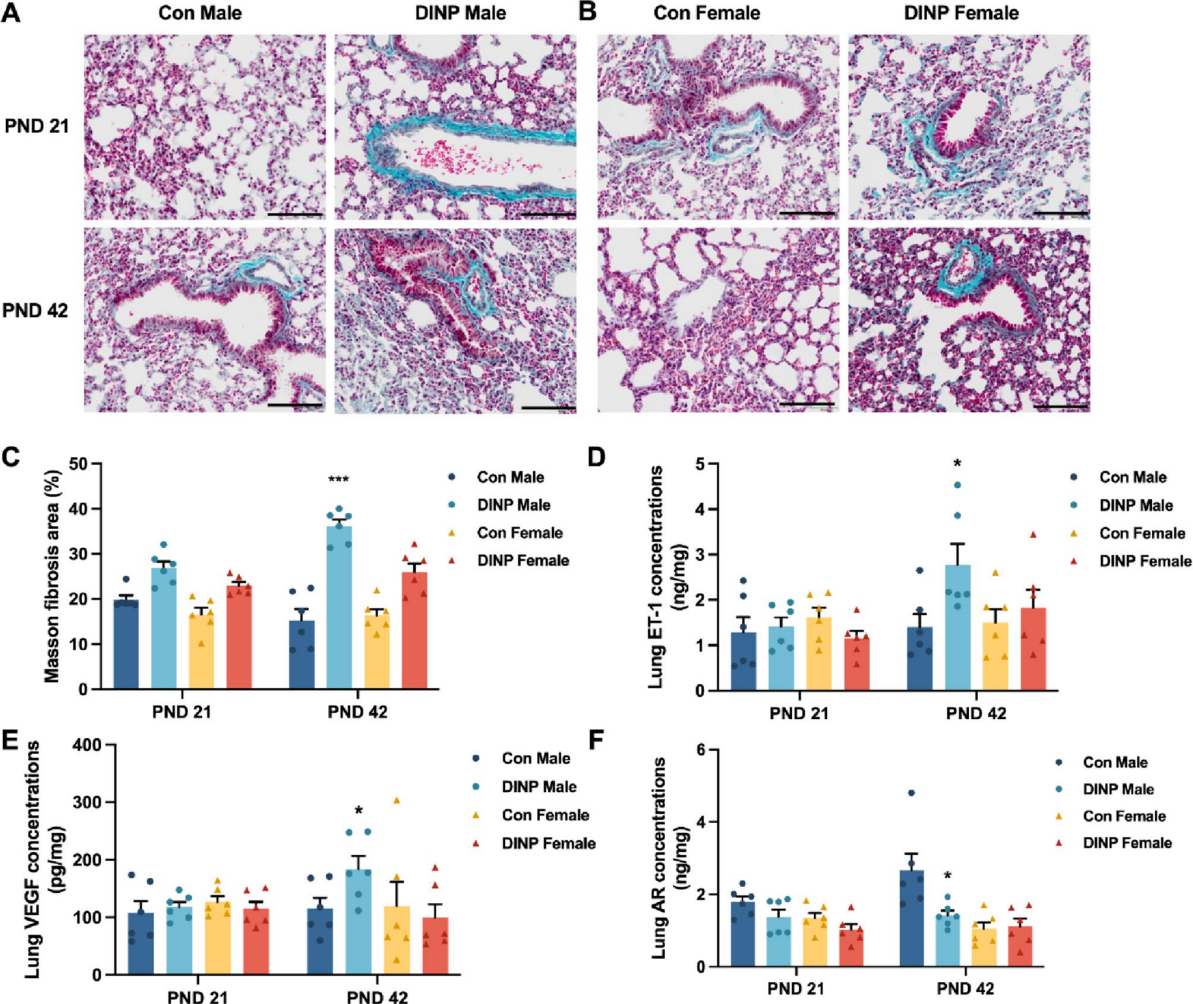
Effects of prenatal DINP exposure on pulmonary fibrosis in offspring. (**A**) Representative images (200×) of Masson–stained lung sections of male offspring on PNDs 21 and 42. Bar = 100 μm. (**B**) Representative images (200×) of Masson–stained lung sections of female offspring on PNDs 21 and 42. Bar = 100 μm. (**C**) Quantification of Masson-stained fibrosis area (*n* = 6 for each group, from two offspring of different dams). (**D**) The ET-1 concentration of lung from offspring on PNDs 21 and 42 (*n* = 6 for each group, from different dams). (**E**) The VEGF concentration of lung from offspring on PNDs 21 and 42 (*n* = 6 for each group, from different dams). (**F**) The AR concentration of lung from offspring on PNDs 21 and 42 (*n* = 6 for each group, from different dams). Data are expressed as mean ± SE. *P < 0.05, ***P < 0.001, indicates significance between different groups. *Con* control; *DINP* prenatal DINP-exposed offspring.

H&E staining showed that there was thickening of alveolar walls in male offspring on PND 42 (Fig. 1D), suggesting that fibrosis might have occurred. Therefore, Masson’s trichrome staining was further employed to confirm the degree of pulmonary fibrosis (Fig. 2A and B). The results revealed that fibrotic lesions in DINP-exposed male offspring were predominantly located in perivascular spaces, forming characteristic collagen-rich “cuffs” around major blood vessels (Fig. 2A). Quantitative analysis confirmed a significant increase in collagen deposition in male offspring on PND 42 (Fig. 2C). In contrast, female offspring exhibited only minimal fibrotic changes (Fig. 2B). The perivascular localization of fibrosis prompted us to assess biomarkers associated with pulmonary vascular dysfunction and pulmonary hypertension. ELISA of the lungs revealed that the levels of ET-1 and VEGF were significantly elevated, specifically in male offspring at PND 42 (Fig. 2D and E). No significant changes were observed in female offspring. To explore the potential mechanism underlying this male-specific susceptibility, we examined the expression of AR (a key sex hormone receptor) in lung tissues. Notably, AR protein expression was significantly downregulated in the lungs of male offspring on PND 42 (Fig. 2F). The localized suppression of AR provides a plausible upstream signal contributing to the sex-dimorphic pathology.

**Fig. 3:**
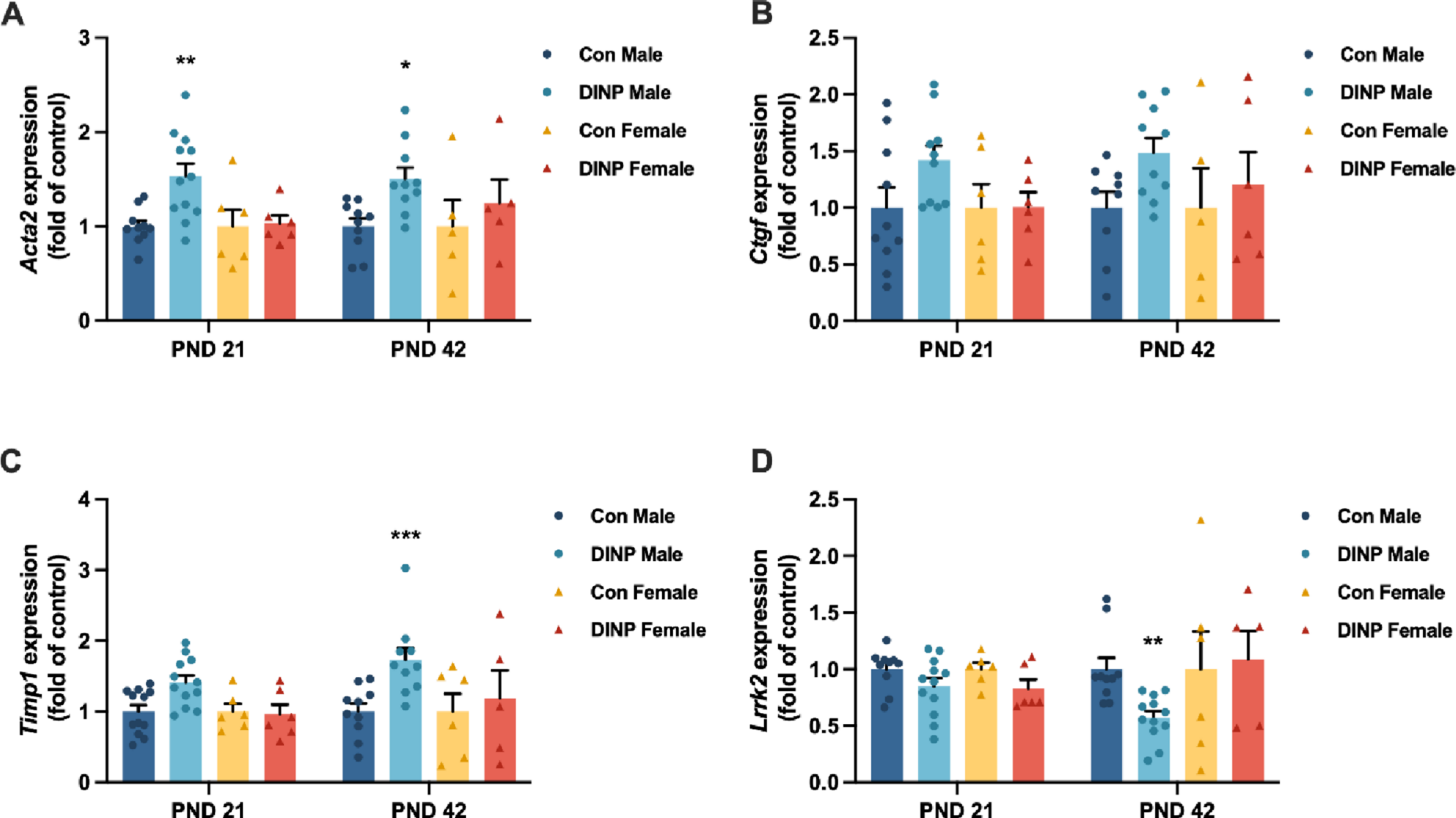
The expression of fibrosis-related genes. (**A**) mRNA expression of *Acta2* (*n* = 5 to 11 for each group, the control was from 7 different dams, and the DINP group was from 6 different dams). (**B**) mRNA expression of *Gtgf* (*n* = 5 to 11 for each group, the control was from 7 different dams, and the DINP group was from 6 different dams). (**C**) mRNA expression of *Timp1* (*n* = 5 to 11 for each group, the control was from 7 different dams, and the DINP group was from 6 different dams). (**D**) mRNA expression of *Lrrk2* (*n* = 5 to 11 for each group, the control was from 7 different dams, and the DINP group was from 6 different dams). Data are expressed as mean ± SE. **P < 0.01, ***P < 0.001, indicates significance between different groups. *Con* control; *DINP* prenatal DINP-exposed offspring.

Furthermore, we examined the mRNA expression of four fibrosis-related genes (*Acta2*, *Timp1*, *Ctgf*, and *Lrrk2*) using RT-qPCR. On PNDs 21and 42, prenatal DINP exposure significantly increased the mRNA expression of *Acta2* in male offspring (Fig. 3A). On PNDs 21and 42, prenatal DINP exposure increased the mRNA expression of *Ctgf* in male offspring, but this increase was not statistically significant (Fig. 3B). On PND 42, prenatal DINP exposure significantly increased the mRNA expression of *Timp1* in male offspring (Fig. 3C), but significantly decreased the mRNA level of *Lrrk2* (Fig. 3D). The results suggest that prenatal DINP exposure induced pulmonary fibrosis in male offspring on PND 42.

### Effects of prenatal DINP exposure on DEGs

**Fig. 4:**
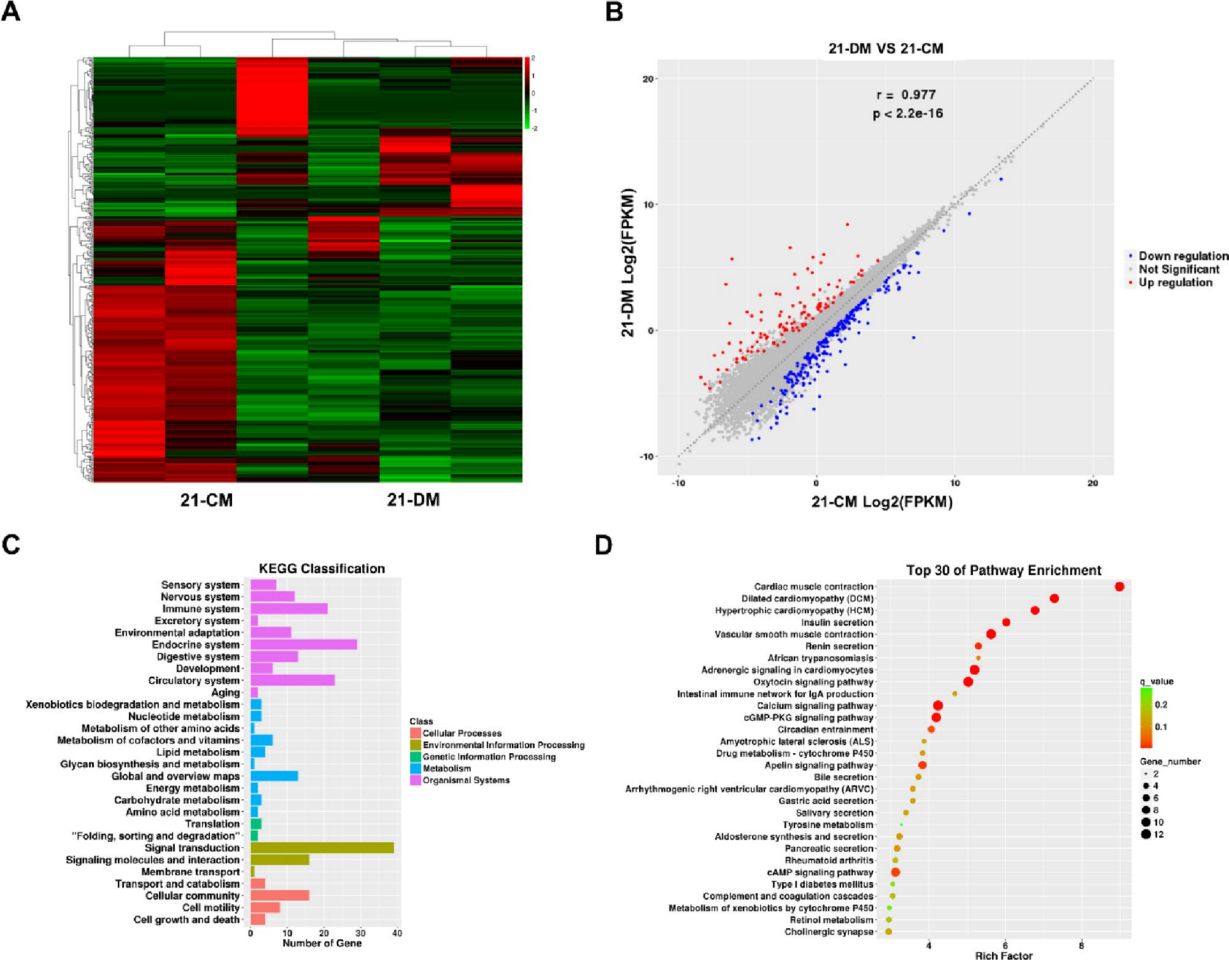
Effects of prenatal DINP exposure on mRNA expression profile and biological functions of DEGs in the lung of male offspring on PND21. (**A**) Heatmap showing expression levels of DEGs. (**B**) Correlation of gene expression between control and prenatal DINP-exposed male offspring. (**C**) KEGG classification of DEGs. (**D**) KEGG enrichment analysis of DEGs (*n* = 3 for each group, from different dams). *DEGs* differentially expressed genes; *KEGG* Kyoto Encyclopedia of Genes and Genomes; *21-CM* control male offspring on PND 21; *21-DM* prenatal DINP-exposed male offspring on PND 21.

**Fig. 5:**
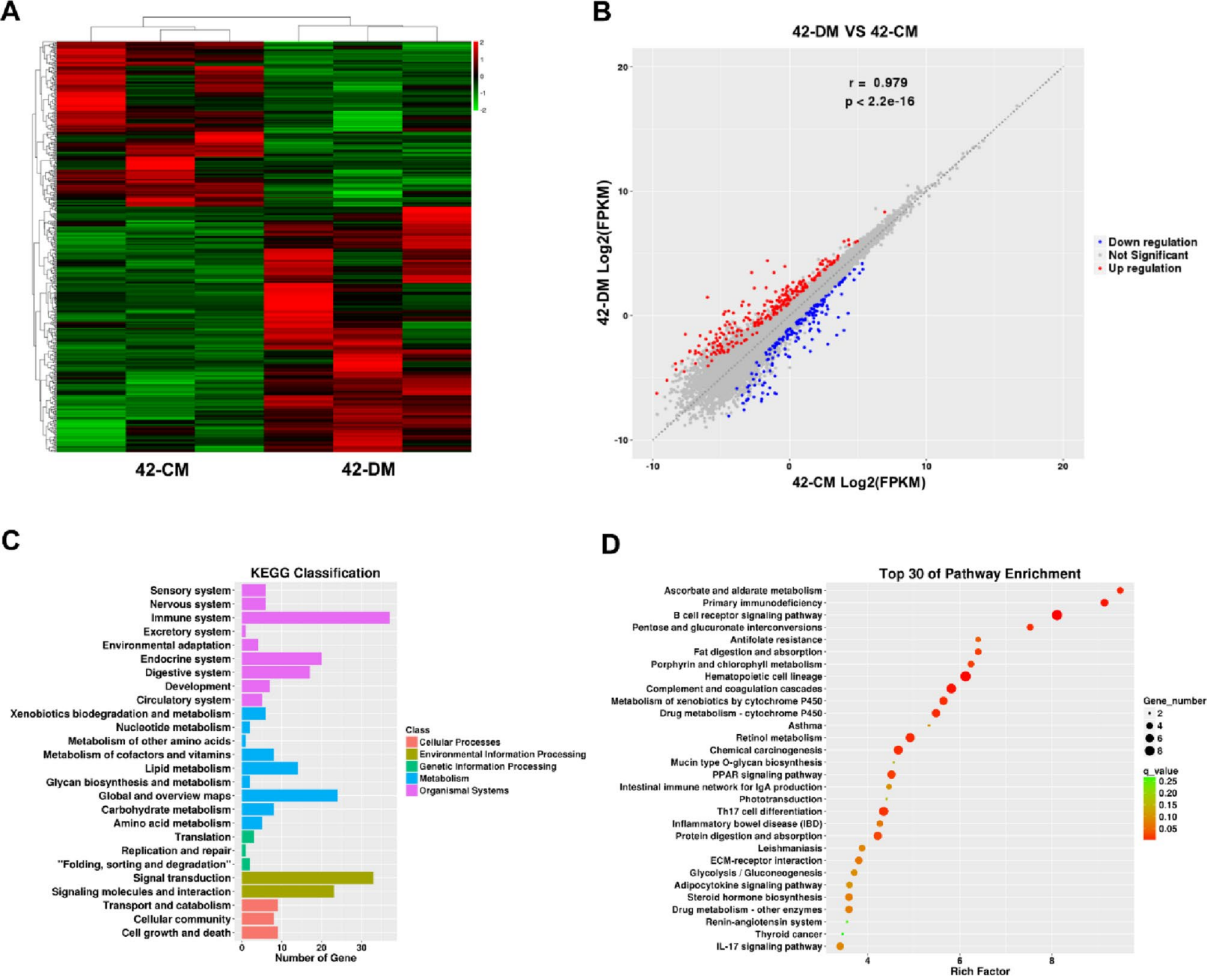
Effects of prenatal DINP exposure on mRNA expression profile and biological functions of DEGs in the lung of male offspring on PND42. (**A**) Heatmap showing expression levels of DEGs. (**B**) Correlation of gene expression between control and prenatal DINP-exposed male offspring. (**C**) KEGG classification of DEGs. (**D**) KEGG enrichment analysis of DEGs (*n* = 3 for each group, from different dams). *DEGs* differentially expressed genes; *KEGG* Kyoto Encyclopedia of Genes and Genomes; *42-CM* control male offspring on PND 42; *42-DM* prenatal DINP-exposed male offspring on PND 42.

Given the observed male-specific pathology and the downregulation of pulmonary AR (Fig. 2F), we performed RNA sequencing to elucidate the underlying global molecular pathways. The results showed that prenatal DINP exposure induced substantial and persistent transcriptional alterations in the lungs of male offspring, with 154 and 159 DEGs at PND 21 and 42, respectively (Fig. 4A and B, 5A and B).

Notably, the enriched pathways evolved from early signaling disturbances to established inflammatory programs. On PND 21, based on the KEGG enrichment analysis^14^, DEGs were primarily enriched in pathways such as calcium signaling (Fig. 4D), potentially representing initial injury responses. On PND 42, the transcriptomic signature shifted decisively towards pathways governing the immune system, with the IL-17 signaling pathway being among the most significantly enriched pathways (Fig. 5D). This temporal shift aligns with the progression from early alveolar simplification to later perivascular fibrosis.

### The roles of IL-17 A and HTR4 in abnormal lung development in male offspring following prenatal DINP exposure

**Fig. 6:**
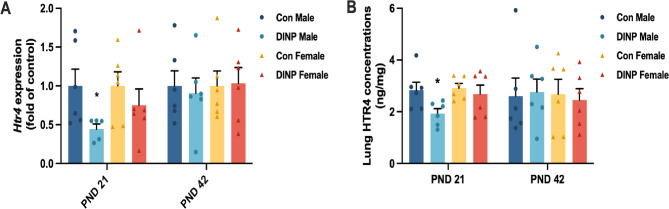
Effects of prenatal DINP exposure on the level of Htr4 in the lungs of offspring. (**A**) mRNA expression of *Htr4* (*n* = 5 to 6 for each group, from different dams). (**B**) The protein expression levels of HTR4 (*n* = 6 for each group, from different dams). Data are expressed as mean ± SE. *P < 0.05, indicates significance between different groups. *Con* control; *DINP* prenatal DINP-exposed offspring.

To further investigate the mechanisms of hypoalveolarization found in male offspring on PND 21 following prenatal DINP exposure, we analyzed the calcium signaling pathway, in which DEGs, including HTR4, TNNC1, and ryanodine receptor 1 (Ryr1), were significantly enriched. The expression of HTR4 protein is prominent in alveolar pneumocytes but low in bronchial epithelial cells, and altered HTR4 expression may be a main cause of pulmonary dysfunction after alveolar dysplasia^15^. Here, we separately examined the expression levels of HTR4 at the genetic and protein levels. The results showed that the mRNA and protein expression levels of HTR4 in male offspring decreased significantly on PND 21 following prenatal DINP exposure (Fig. 6A and B). In contrast, we did not observe a similar phenomenon in female offspring. Therefore, prenatal DINP exposure decreased the expression of HTR4 and caused hypoalveolarization in male offspring on PND 21.

**Fig. 7:**
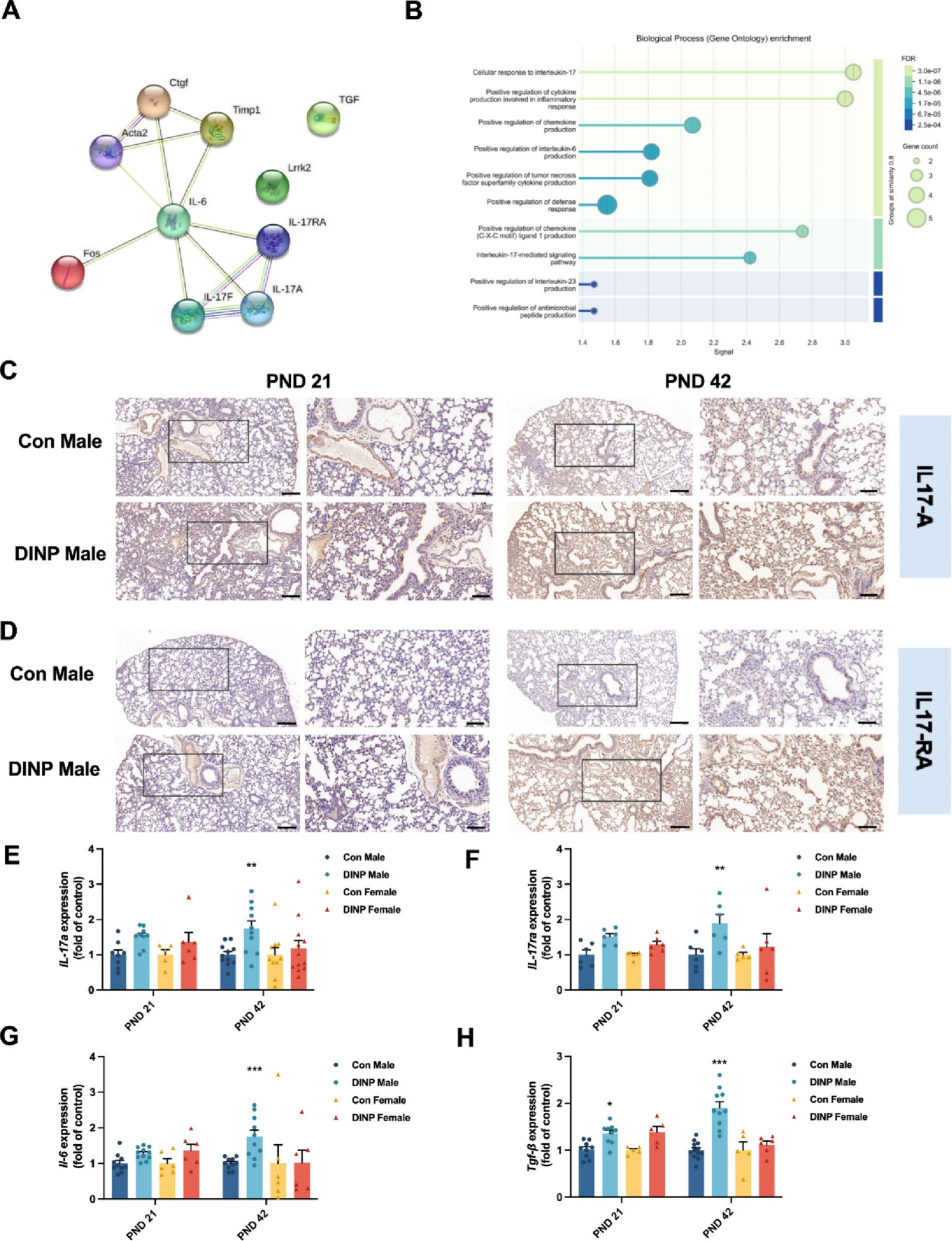
Involvement of the IL-17 signaling pathway in prenatal DINP expose-induced sex-dependent pulmonary fibrosis in offspring on PND 42. (**A**) PPI network of the IL-17 signaling pathway and pulmonary fibrosis-related genes. (**B**) The functional enrichment of PPI networks. (**C**) Representative IHC images (left: 100×; right: 200×) of lung sections of male offspring stained with IL-17A on PNDs 21 and 42 (2 males from different litters per time point). (**D**) Representative IHC images (left: 100×; right: 200×) of lung sections of male offspring stained with IL-17RA on PNDs 21 and 42 (2 males from different litters per time point). (**E**) mRNA expression of *Il-17a* (*n* = 5 to 11 for each group, the control was from 7 different dams, and the DINP group was from 6 different dams). (**F**) mRNA expression of *Il-17ra* (*n* = 5 to 6 for each group, from different dams). (**G**) mRNA expression of *Il-6* (*n* = 6 to 11 for each group, the control was from 7 different dams, and the DINP group was from 6 different dams). (H) mRNA expression of *Tgf-β* (*n* = 5 to 11 for each group, the control was from 7 different dams, and the DINP group was from 6 different dams). Data are expressed as mean ± SE. *P < 0.05, **P < 0.01, ***P < 0.001, indicates significance between different groups. *Con* control; *DINP* prenatal DINP-exposed offspring.

IL-6 and TGF-β are profibrotic cytokines that can induce pulmonary fibrosis^16–18^. On PND 42, the DEGs enriched in the IL-17 signaling pathway included IL-17A, IL-17F, and Fos. So we tried to elucidate the association between the IL-17 signaling pathway and pulmonary fibrosis in male offspring. To obtain the protein-protein interaction (PPI) network using the String 12.0 database (https://stri-ng-db.org), IL-17A, IL-17F, Fos, TGF-β, IL-6, and fibrosis-related genes (*Acta2*, *Timp1*, *Ctgf* and *Lrrk2*) were loaded, and the species and the minimum required interaction score were set as “Mus musculus” and high confidence (0.700), respectively. We found that IL-6 was the main protein linking the IL-17 signaling pathway and fibrosis-related genes (Fig. 7A). Moreover, the functional enrichment of PPI networks was visualized (Fig. 7B). The result showed that four biological processes, including cellular response to IL-17, positive regulation of chemokine production, positive regulation of cytokine production involved in inflammatory response, and positive regulation of IL-6 production, had low false discovery rate (FDR) and a large number of enriched genes. It’s reported that IL-17F has at least 100 times lower affinity for IL-17RA compared to IL-17A^19^. Next, we detected the expression of *Il-17a*, *Il-17ra*, *Il-6*, and *Tgf-β*. The mRNA levels of *Il-17a* and *Il-17ra* in the lungs of male offspring were significantly higher in the prenatal DINP exposure group than in the control group on PND 42 (Fig. 7E and F), which were validated by staining for anti-IL-17A and anti-IL-17RA in lung sections (Fig. 7C and D). The results of female offspring are provided in Fig. S1 in the SM. The mRNA levels of *Il-6* and *Tgf-β* in the lungs of male offspring were significantly higher in the prenatal DINP exposure group than in the control group on PND 42 (Fig. 7G and H). In contrast, the similar phenomenon was not observed in female offspring. IL-17A can bind to the receptor, and activate the adaptor protein ACT1 and nuclear factor-kappaB (NF-κB), leading to increased transcription of *Il-6*^20^. Therefore, prenatal DINP exposure might be associated with sensitivity related to IL-17A in male offspring, which needs to be further explored in future studies.

## Discussion

Here, we studied the impact of prenatal DINP exposure on lung development in offspring. The results showed that prenatal DINP exposure affected alveolar development in male offspring on PND 21, and induced pulmonary fibrosis and lung inflammation in male offspring on PND 42. However, we did not find that prenatal DINP exposure affected lung development in female offspring, suggesting that prenatal DINP exposure affected offspring lung development in a sex-dependent manner.

Alveologenesis occurs postnatally in mice^21^, which is regulated by several critical mediators, such as Eln, Pdgfr-α, and Loxl1. Inactivation of Pdgfr-α arrests alveologenesis, leading to BPD. Moreover, the absence of Pdgfr-α disrupts the expression of elastogenic genes, such as Lox, Fbn, and Fbln families^21^. The mRNA and protein levels of lysyl oxidases (Lox, Lox11, and Lox12), as well as their activities, increase in the lungs of newborn mice with BPD or at risk for BPD following oxygen injury^22^. Excess activities of lysyl oxidases can inhibit the remodeling of the extracellular matrix (ECM), thereby affecting pulmonary alveolarization^11^. Eln is expressed at high levels during secondary septation (PND5 - PND15) but down-regulated rapidly after alveolarization^23^. Here, we found that the level of *Pdgfr-α* was significantly reduced and the levels of *Eln* and *Loxl* increased significantly in male offspring on PND 21 after prenatal DINP exposure, indicating that decreased *Pdgfr-α* expression could affect the expression of *Loxl*, thereby impeding the formation of alveoli and increasing the expression of *Eln*.

Damage to the alveoli can lead to structural lung diseases, such as chronic obstructive pulmonary disease (COPD), pulmonary fibrosis, and emphysema^24^. Here, the Masson-stained lung sections revealed that prenatal DINP exposure caused fibrotic lesions in the lungs of male offspring on PND 42. Fibrotic remodeling appears more pronounced around major blood vessels which is an important finding and suggests a potential link to pulmonary vascular remodeling and hypertension. To confirm this link, we measured the expression of two key vascular mediators, including ET-1 and VEGF, in lung tissues. We found that ET-1 and VEGF protein levels significantly increased in the lungs of male offspring on PND42 following prenatal DINP exposure. These two mediators are critically involved in vasoconstriction, vascular smooth muscle cell proliferation, and angiogenesis, playing central roles in the pathogenesis of pulmonary hypertension. The perivascular fibrosis, coupled with elevated ET-1 and VEGF, suggests that prenatal DINP exposure-induced developmental insult may not only cause parenchymal fibrosis but also predispose to pulmonary vascular dysfunction. To verify structural abnormalities, the expression of four fibrosis-related genes (A*cta2*, *Timp1*, *Ctgf*, and *Lrrk2*) was determined. ACTA2 is an actin isoform facilitating cell contraction and migration and is considered a hallmark of myofibroblasts^25^. TIMP1 is highly expressed during fibrosis, and its overexpression can cause ECM accumulation, thus promoting fibrosis progression^26,27^. CTGF exerts a broad impact on cell migration, adhesion, and proliferation, making it a critical factor in fibrotic diseases^28^. LRRK2 is broadly expressed in various cells and participates in many processes, such as vesicle trafficking, autophagy, and neuronal plasticity^28,29^. Previous studies show that LRRK2 expression is remarkably down-regulated in fibrotic lungs^30^. Here, we found that the level of *Lrrk2* decreased significantly and the levels of *Acta2*, *Timp1*, and *Ctgf* were significantly elevated in male offspring, but not female offspring, on PND 42 after prenatal DINP exposure. Similarly, DINP increases airway hyperresponsiveness, airway inflammation, and pulmonary fibrosis^5^. Epidemiological studies indicate that prenatal exposure to environmental pollutants (including DINP) is associated with childhood asthma^31^. Prenatal DINP exposure can cause adverse birth outcomes and affect adolescent development^32,33^. Here, our results further demonstrated that male offspring were more sensitive to pulmonary fibrosis outcomes caused by prenatal DINP exposure.

Moreover, we used mRNA-Seq to unravel the possible mechanism. On PND 21 following prenatal DINP exposure, DEGs were mainly enriched in the calcium signaling pathway, one of the most important intracellular signaling pathways. We focused on *Htr4*, one of the DEGs involved in this pathway, and found that the mRNA level of *Htr4* was significantly reduced in male offspring on PND 21 after prenatal DINP exposure, but not in female offspring. High expression of *Htr4* is found in the central nervous system, especially in the limbic system associated with anxiety, depression, learning and memory, and behavior^34^. *Htr4* is also slightly expressed in lung airway smooth muscle and epithelial cells^35,36^. Interestingly, differential expression of *Htr4* is found at diverse stages of lung development, with the most predominant expression in alveolar pneumocytes^15^. In HTR4-deficient mice, both whole lung resistance and airway resistance increase at baseline^37^. Therefore, HTR4 might be responsible for sex differences in hypoalveolarization on PND 21. Moreover, we found that DEGs were predominantly enriched in the primary immunodeficiency and IL-17 signaling pathway on PND 42. IL-17 A is a predominant inflammatory cytokine secreted by T helper 17 (Th17) cells^38^. The IL-17A/IL-17RA system regulates the mitogen-activated protein kinase and NF-κB signaling pathways, and the activation of IL-17RA triggers downstream signaling cascades, thereby releasing pro-inflammatory cytokines (such as IL-6) and CXC chemokines to further enhance neutrophil-mediated immunity^39^. IL-17 A can induce the epithelial-mesenchymal transition in epithelial cells by producing TGF-β, and it can also directly stimulate fibroblasts and fibrocytes^40^. In addition, IL-6 and TGF-β are crucial profibrotic cytokines in inducing pulmonary fibrosis. In this study, on PND 42 following prenatal DINP exposure, the mRNA levels of *Il-17a*, *Il-17ra*,* Il-6* and *Tgf-β* increased significantly in male offspring, but not in female offspring. Lung AR signaling can alleviate inflammation and improve the outcome of influenza in mice^41^. Our findings establish a strong association between prenatal DINP exposure-induced AR downregulation, elevated IL-17A expression, and more severe fibrotic remodeling in male offspring. However, the definitive causal relationships within this proposed AR–IL-17 pathway require validation through future mechanistic studies.

It should be noted that the level of DINP exposure used in this study reflects a toxicological dose rather than a physiological one. This experimental design was chosen to elucidate potential mechanisms and pathological outcomes under conditions of significant exposure. While this approach helps to identify clear biological effects and signaling pathways, further studies using environmentally relevant or lower doses are needed to assess the real-world public health implications of prenatal DINP exposure. In addition, the present study used a wild-type mouse strain and did not model a known genetic component of susceptibility to pulmonary fibrosis caused by environmental stress. This might be a limitation of the current study design, and future studies should use a genetically engineered mouse model to further elucidate the mechanisms underlying the pulmonary fibrosis in male offspring following DINP exposure, as well as the role of IL-17A.

## Conclusion

Collectively, our work demonstrated that prenatal DINP exposure induced hypoalveolarization and pulmonary fibrosis in male offspring, but only reversible changes in alveolarization in female offspring. Compared to female offspring, male offspring showed higher susceptibility to lung injury induced by prenatal DINP exposure. Moreover, HTR4 and IL-17A might play critical roles in the induction of hypoalveolarization and pulmonary fibrosis following prenatal DINP exposure. The findings offer experimental evidence for the development of pulmonary dysplasia in male offspring by prenatal DINP exposure.

## Supplementary Information

Below is the link to the electronic supplementary material.

Supplementary Material .

## Data Availability

The datasets generated and/or analysed during the current study are available in the [GEO] repository, [https://www.ncbi.nlm.nih.gov/geo/query/acc.cgi?acc=GSE315505]. In addition, the data related to this study have been included as supplementary material and are available as part of the manuscript submission.
