# Supplementary material for: Prenatal diisononyl phthalate exposure induces the development of pulmonary dysplasia in offspring
Source: Sci Rep. 2026 May 3;16:20425. doi: 10.1038/s41598-026-50939-8 (PMC13328291; doi:10.1038/s41598-026-50939-8)
Supplement: Supplementary file 1 — Supplementary Material . [file 41598_2026_50939_MOESM1_ESM.docx]

**Cover Sheet**

| Manuscript Title | Prenatal diisononyl phthalate exposure induces the development of pulmonary fibrosis in male offspring |
| --- | --- |
| Complete List of Authors | Dan Li, Chengyu Zhao, Fu Xu, Yao Yuan, Sihan Zhang, Yuanyuan Tian, Meiqiong Wu* |
| Total Pages | 3 |
| Total Texts | 1 |
| Total Figures | 1 |

**Text**

**S1: Dose selection criteria**

To clarify the scientific rationale for selecting this exposure level, particularly in the context of early-life sensitivity during developmental windows, we provide the following explanation:

According to the EU Risk Assessment Report (EU RAR), the tolerable daily intake (TDI) for DINP in humans is 0.15 mg/kg/day (Huang et al. 2019). Using the body surface area conversion factor recommended by the U.S. Food and Drug Administration (FDA) for humans to mice (1:12.3), the corresponding mouse-equivalent TDI is calculated as 0.15 × 12.3 = 1.845 mg/kg/day. Although the dose used in our study (100 mg/kg/day) is approximately 54 times this value, its selection was based on several scientific considerations:

The European Chemicals Agency (ECHA, 2010) identified a no-observed-adverse-effect level (NOAEL) of 4.8 mg/kg/day for DINP in mice based on hepatic toxicity endpoints (Yang et al. 2021).

Our study focuses specifically on hepatic toxicity during early developmental stages. Previous studies have shown that perinatal exposure to DINP at doses as low as 15 mg/kg/day can induce measurable alterations in hepatic metabolic pathways in neonatal mice, indicating that early life may represent a particularly sensitive window for phthalate-induced liver metabolic disturbances (Neier et al. 2020).

Human studies report amniotic fluid concentrations of phthalates ranging from <LOD to 100.6 ng/mL. In rodents, oral exposure to 11 mg/kg/day phthalates resulted in amniotic fluid concentrations of 68 ng/mL (Neier et al. 2020). Based on this relationship, the 100 mg/kg/day dose used in our study is estimated to produce an amniotic fluid concentration of approximately 618 ng/mL. Considering that developmental toxicity studies typically employ a 5–10-fold exposure margin to account for interspecies and intraspecies variability, and to link observed toxic effects in animals to potential human exposure levels, the dose selected in this study is scientifically justified within the context of developmental toxicology research. Based on the above research, we have determined the doses of DINP to be 100 mg/kg/day.

**S2: Immunohistochemical (IHC) staining.**

Prior to the IHC staining, paraffin sections were deparaffinized by soaking in xylene twice for 10 min each, and then soaked in graded concentrations of ethanol (100%, 95%, 75% and 50%) for 5 min intervals. Finally, the sections were washed in distilled water for 5 min, soaked in PBS for 5 min, and immersed in diluted citrate buffer (1 M pH 6.0, Beijing Biosynthesis Biotechnology Co., Ltd., Beijing, China; citrate buffer: distilled water = 1: 100) for 15 min in a 95 °C water bath for antigen retrieval.

IHC staining was conducted according to the manufacturer’s instructions (Histostain^TM^-plus kit and primary antibody were purchased from Beijing Biosynthesis Biotechnology Co., Ltd., Beijing, China). Briefly, after antigen retrieval, the sections were first treated with 3% H_2_O_2_ in methanol (Histostain^TM^-plus kit) for 15 min at room temperature (RT) to quench the endogenous peroxidase activity. Then, the sections were blocked with Buffer A of the Histostain^TM^-plus kits for 18 min at RT, followed by incubation with anti-IL-17A antibody (1:200, cat. no. ER1902-37, Huabio) and anti-IL-17RA antibody (1:200, cat. no. Ab218249, Abcam) overnight at 4 °C. On the following day, the sections were incubated with Buffer B of the Histostain^TM^-plus kits for 18 min at RT after being washed with PBS 3 times. The sections were subsequently washed with PBS 3 times and incubated with Buffer C of the Histostain^TM^-plus kit for 18 min at RT. After being washed with PBS 3 times, the sections were then incubated in diaminobenzidine (DAB) solution for 10 min at RT. Finally, the sections were counterstained with hematoxylin and observed under a light microscope.


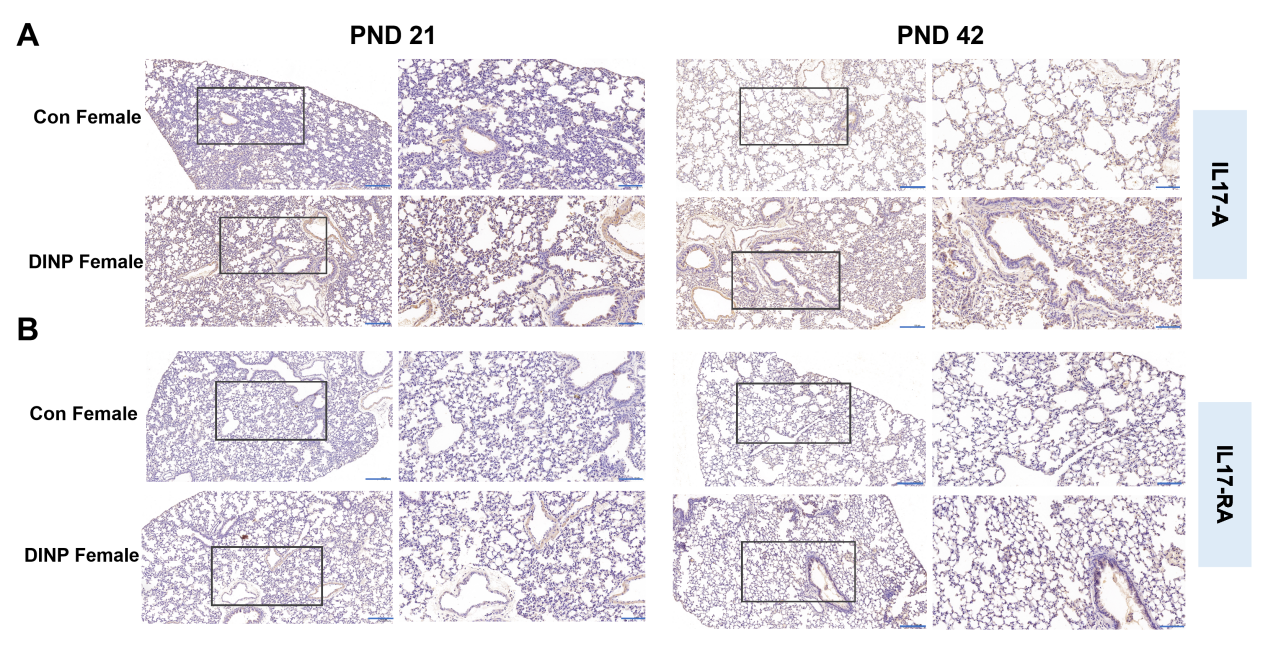


Fig. S1: IHC staining results of female offspring. (A) Representative IHC images (left: 100×; right: 200×) of lung sections of female offspring stained with IL-17A on PNDs 21 and 42 (2 females from different litters per time point). (B) Representative IHC images (left: 100×; right: 200×) of lung sections of female offspring stained with IL-17RA on PNDs 21 and 42 (2 females from different litters per time point). Con: control; DINP: prenatal DINP-exposed offspring.

**References**

Huang, Y., Sun, F., Tan, H., Deng, Y., Sun, Z., Chen, H., t al., 2019. DEHP and DINP Induce Tissue- and Gender-Specific Disturbances in Fatty Acid and Lipidomic Profiles in Neonatal Mice: A Comparative Study. Environmental science & technology. 53(21), 12812-12822.

Neier, K., Montrose, L., Chen, K., Malloy, M. A., Jones, T. R., Svoboda, L. K., et al., 2020. Short- and long-term effects of perinatal phthalate exposures on metabolic pathways in the mouse liver. Environmental Epigenetics. 6(1),dvaa017.

Yang, Y., Sun, F., Chen, H., Tan, H., Yang, L., Zhang, L., et al., 2021. Postnatal exposure to DINP was associated with greater alterations of lipidomic markers for hepatic steatosis than DEHP in postweaning mice. Science of The Total Environment. 2758, 143631.
